# Ten Topics to Get Started in Medical Informatics Research

**DOI:** 10.2196/45948

**Published:** 2023-07-24

**Authors:** Markus Wolfien, Najia Ahmadi, Kai Fitzer, Sophia Grummt, Kilian-Ludwig Heine, Ian-C Jung, Dagmar Krefting, Andreas Kühn, Yuan Peng, Ines Reinecke, Julia Scheel, Tobias Schmidt, Paul Schmücker, Christina Schüttler, Dagmar Waltemath, Michele Zoch, Martin Sedlmayr

**Affiliations:** 1 Institute for Medical Informatics and Biometry Faculty of Medicine Carl Gustav Carus Technische Universität Dresden Dresden Germany; 2 Center for Scalable Data Analytics and Artificial Intelligence Dresden Germany; 3 Core Unit Data Integration Center University Medicine Greifswald Greifswald Germany; 4 Department of Medical Informatics University Medical Center Goettingen Germany; 5 Department of Systems Biology and Bioinformatics University of Rostock Rostock Germany; 6 Institute for Medical Informatics University of Applied Sciences Mannheim Mannheim Germany; 7 Central Biobank Erlangen University Hospital Erlangen Friedrich-Alexander-Universität Erlangen-Nürnberg Erlangen Germany; 8 Department of Medical Informatics University Medicine Greifswald Greifswald Germany

**Keywords:** medical informatics, health informatics, interdisciplinary communication, research data, clinical data, digital health

## Abstract

The vast and heterogeneous data being constantly generated in clinics can provide great wealth for patients and research alike. The quickly evolving field of medical informatics research has contributed numerous concepts, algorithms, and standards to facilitate this development. However, these difficult relationships, complex terminologies, and multiple implementations can present obstacles for people who want to get active in the field. With a particular focus on medical informatics research conducted in Germany, we present in our Viewpoint a set of 10 important topics to improve the overall interdisciplinary communication between different stakeholders (eg, physicians, computational experts, experimentalists, students, patient representatives). This may lower the barriers to entry and offer a starting point for collaborations at different levels. The suggested topics are briefly introduced, then general best practice guidance is given, and further resources for in-depth reading or hands-on tutorials are recommended. In addition, the topics are set to cover current aspects and open research gaps of the medical informatics domain, including data regulations and concepts; data harmonization and processing; and data evaluation, visualization, and dissemination. In addition, we give an example on how these topics can be integrated in a medical informatics curriculum for higher education. By recognizing these topics, readers will be able to (1) set clinical and research data into the context of medical informatics, understanding what is possible to achieve with data or how data should be handled in terms of data privacy and storage; (2) distinguish current interoperability standards and obtain first insights into the processes leading to effective data transfer and analysis; and (3) value the use of newly developed technical approaches to utilize the full potential of clinical data.

## Introduction

Digital health care information, as opposed to analog information, empowers clinicians, researchers, and patients with a wealth of information aiming to improve diagnosis, therapy outcome, and clinical care in general. According to Wyatt and Liu [1], medical informatics is the study and application of methods to improve the management of patient data, clinical knowledge, population data, and other information relevant to patient care and community health. Medical informatics can be seen as the subset of health informatics that is focused on clinical care, while the latter encompasses a wider range of applications. However, knowing, integrating, and using current computational technologies bears numerous pitfalls, limitations, and questions [2]. To shed light on current standards, applications, and underlying technologies, we present 10 topics to get started in the field of medical informatics research. Our key objective here was to improve interdisciplinary communication among stakeholders (eg, clinicians, experimental researchers, computer scientists, students, patient representatives), thereby bringing everyone on the same page of state-of-the-art medical informatics practices. In particular, improved interdisciplinary communication is essential in real-world problems and can be motivated by the following aspects:

- Advancing open research: Open collaboration between parties from different disciplines can lead to new research questions, innovative approaches, and novel discoveries [3].
- Bridging knowledge domains: Interdisciplinary communication can stimulate novel solutions, allowing researchers to gain a more comprehensive understanding of a specific problem or phenomenon [4], or can improve clinical decision-making [5].
- Addressing complex problems: Complex problems, such as the latest disease outbreak, require input from multiple domains to be comprehensively understood. Here, interdisciplinary communication is one key aspect to pinpoint the root causes and develop effective solutions [6].
- Promoting scientific inclusivity and diversity: Interdisciplinary communication was recently shown to foster diversity and inclusivity in science, by bringing together researchers from different backgrounds, cultures, and perspectives [7,8].

## Methods

Here, we describe in detail how the initial topics have been selected from the literature and what design principles and structure each topic follows. A brief outline of the utilized methods for topic dissemination and an exemplary embedding into an educational training program are also presented.

### Topic Selection

**Figure 1:**
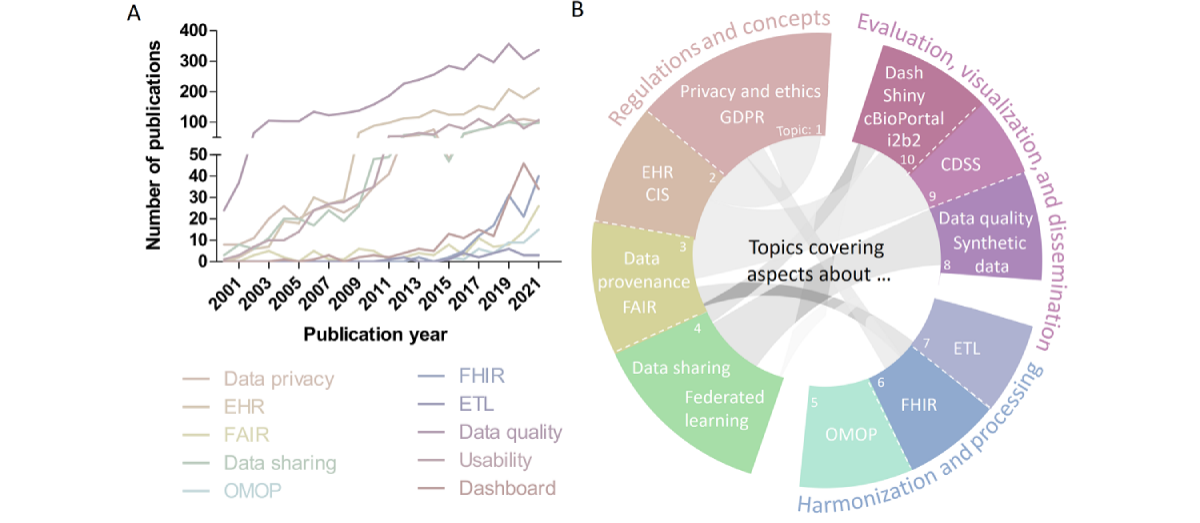
Schematic summary and representation of the presented topics: (A) brief literature screening (title-abstract-keywords) for published manuscripts between 2000 and 2021, and the y-axis gap provides improved visibility of the less-occurring keywords; (B) most common topic terminologies, keywords (color-coded sections), and potential connections (grey) among topics in the medical informatics research domain. CDSS: clinical decision support system; CIS: clinical information system; EHR: electronic health record; ETL: extract, transform, and load; FAIR: findable, accessible, interoperable, reusable; FHIR: Fast Healthcare Interoperability Resources; GDPR: General Data Protection Regulation; i2b2: Informatics for Integrating Biology and the Bedside; OMOP: Observational Medical Outcomes Partnership.

The initial topics were defined based on current developments in the health informatics field and an increasing number of published manuscripts between 2000 and 2021 (based on title-abstract-keyword screening in Scopus using the keywords “Health” AND “Informatics” AND “domain”) in the respective subdomains (Figure 1A). After a first definition of the specific topics, these were critically revised by internal and external domain experts, as well as scientists previously not familiar with medical informatics research.

### Topic Design

The initial number of important topics and keywords exceeded the anticipated number of 10 topics, which found inspiration from the “Ten Simple Rules” collection in PLOS Computational Biology [9]. This is why the authors merged the most matching terms topic wise into groups. These groups finally produced topics that represent the broad range of the medical informatics domain in 3 main concepts, namely “Regulations and concepts,” “Harmonization and processing,” and “Evaluation, visualization, and dissemination” (Figure 1B). Figure 1B also shows the initial keywords for each individual topic, as well as potential cross references between topics, which are highlighted in grey. The following sections provide important “do's and don'ts,” practical hints, and best practice guidelines. Further in-depth resources and practical tutorials will provide basic introductions to the referred domains. Kohane et al [10] already showed the importance of such clarifying introductions. This work extends the initial study and, in addition, provides detailed examples from the German national Medical Informatics Initiative (MII) [11].

All topics were divided into 3 parts to improve comprehension by the readers:

1. Introduction: Background definitions for the specific context that motivated the topic
2. Insight: Practical context to get started, including how to avoid pitfalls, state current limitations, and address current challenges
3. Impact: Take home message and useful resources and best practices to deepen knowledge about the topic

### Topic Utilization, Extension, and Embedding

Since it is of the utmost importance to keep the content current and as versatile as possible, we initiated an online resource at GitHub, in which contributions are highly emphasized [12]. Here, keywords and the corresponding literature are collected to allow for swift extension of the currently presented literature body in this article. In addition, the introduction of novel important topics that are not covered in this article might be included. To additionally demonstrate the practicability and adaptability of our proposed topic content, we exemplarily present how these can be embedded in higher education training and share external, introductory hands-on material (Table 1).

## Results

### Regulations and Concepts

#### Topic 1: Privacy and Ethics—“Data Privacy and Ethics Are the Most Important Assets in the Clinical Domain.”

##### Introduction

Health information is sensitive and hence needs to be highly protected and should not be generously shared. Sharing regulations and data privacy matters are defined in the European General Data Protection Regulation (GDPR) [13]. The implementation of the GDPR is an *ongoing* process as the quickly evolving technology, data, and scientific practices demand continuous improvement, which include periodic adaptations of the technical and legal aspects [14,15]. In terms of ethics and with the rise of novel technologies, like artificial intelligence (AI), the possible re-identification of data, such as images and genomic information, is a major concern [16,17].

##### Insight

Anonymization is one important way to keep data private. It can also be achieved for high-dimensional data by changing patient-specific identifiers through removal, substitution, distortion, generalization, or aggregation [18]. In contrast, data pseudonymization is another de-identification procedure by which personally identifiable information fields within a data record are replaced by one or more artificial identifiers or pseudonyms [19]. To overcome the paucity of annotated medical data in real-world settings and (fully) save the patients’ anonymity, *synthetic data* generation is used to increase the diversity in data sets and to enhance the robustness and adaptability of AI models [20]. To conform with ethical regulations in a research context, medical data are only available in a highly controlled manner and according to strict procedures. New concepts, such as “systemic oversight” [21] or “embedded ethics” [22], might be needed to tackle the new data-driven developments around “medical big data” and AI in health care. To engage with the adoption of broad consent, systemic oversight was suggested as an approach, in which mechanisms like auditing mechanisms, expert advice, and public engagement initiatives (among others) should be adapted as additional layers to the newly arising ecosystem of health data [21]. Recently, embedded ethics was jointly suggested by ethicists and developers to address ethical issues via an iterative and continuous process from the outset of development, which could be an effective means of integrating robust ethical considerations into practical development [22]. A digital representation of information encoded in signed consent forms is needed to facilitate common data use and sharing, as already implemented in an MII informed consent template [23].

##### Impact

As a researcher in medical informatics, it is inevitable to be informed and knowledgeable about the fact that patients own their medical records and any use of those data requires great care. In Germany, health care providers can only use the data for first medical use. Secondary use, like research, needs to be approved by either broad or individual consent, which can be made available via the electronic health record (EHR). In addition to digitization efforts, it is still a considerable hurdle to convince patients to make their data available for medical research because personal skepticism commonly makes the entire data acquisition process more difficult [24]. Here, well-received external communication, transparency, and increased awareness are necessary for substantial improvements. In general, it is a balance between privacy, patient needs, and the use of data for the common good versus economic interests [25]. In particular, one should be aware of the specific legal regulations that apply within the country and additionally get in touch with the relevant data protection departments. Following this, a plan for infrastructure that meets these regulations and that contains, for example, a trustee for the electronic recording of patient consent and anonymization or direct pseudonymization processes to collect the data needs to be developed. Risk assessments for potential data leakage, approvals by ethics committee, as well as consultation with a data protection officer are essential considerations to further assure data security.

#### Topic 2: EHR and Clinical Information Systems—“Get to Know Your Clinical Information System to Understand the Required Data.”

##### Introduction

Hospitals run clinical information systems (CIS) to collect, store, and alter clinical data about patients. A CIS, independent of the specialization and specific vendor, covers many clinical subdomains and integrates patient-related data to support doctors in their daily routine. Without a doubt, medical data are only useful if meaningful information can be derived from them. This requires high-quality data sets, seamless communication across IT systems, and standard data formats that can be processed by humans and machines [2]. Typical challenges in clinical IT implementations, especially for patient recruitment systems, were recently evaluated by Fitzer et al [26] for 10 German university hospitals, including requirements for data, infrastructure, and workflow integration. The implementation of an EHR, including an individual's medical data in a bundled form, into the CIS is a key aspect to prevent low reliability and poor user-friendliness of EHRs, which has recently been shown to affect time pressure among medical staff [27]. For example, in Scandinavia, the United States, and the United Kingdom, the Open Notes initiative [28] facilitates patients’ access to EHRs and health data sharing via “PatientsKnowBest*”* to give health care professionals and families direct access to medical information [29].

##### Insight

An EHR is used primarily for the purposes of setting objectives and planning patient care, documenting the delivery of care, and assessing the outcomes of care [30]. EHRs have so far consisted of unstructured, narrative text as well as structured, coded data. Thus, it will be necessary to implement more systematic terminologies and codes so that the data contained in these records can be reused in clinical research, health care management, health services planning, and government reporting in an improved manner [31,32]. Since the domain of medical informatics is rather new, there are many possibilities for software solutions to improve EHR-related issues [33]. Exemplary for the EHR domain, the Systematized Nomenclature of Medicine and Clinical Terms (SNOMED CT) is utilized to develop comprehensive high-quality clinical content [34]. It provides a standardized way to represent clinical phrases captured by the clinician and enables automatic interpretation of these, which is showcased in a “five-step briefing” [35]. Interestingly, the number of annual publications on this subject has decreased since 2012. However, the need for a formal semantic representation of free text in health care remains, and automatic encoding into a compositional ontology could be a solution [36]. In terms of usability and user acceptance, evaluations and improvements of EHRs and clinical decision support systems (CDSS) are currently ongoing [37], for which already well-received examples can be attributed to CeoSYS [38] or the IPSS-M Risk Calculator [39]. Moreover, the actions of patients directly contributing to their own EHR records are also being evaluated. The study by Klein et al [40] indicates that such an approach facilitates the development of individual solutions for each patient, which in turn requires a flexible EHR during the course of a treatment process. Additionally, it was argued that data incorporation via different devices can also facilitate the convenient utilization of the application and, hence, may increase secondary use.

##### Impact

Modern CIS support the interaction by doctors and patients with the recorded patient data (eg, using the EHR or patient portals, eHealth platforms). It is important to understand the basic architecture, especially challenges [26], of the hospital IT infrastructure to know where data are located and how they can be retrieved and integrated. Major improvements can be made when supporting international standards for data exchange. Beyond standard EHR, this includes interoperability standards like Fast Healthcare Interoperability Resources (FHIR; see Topic 6) and standard data models like the Observational Medical Outcomes Partnership (OMOP; see Topic 7). These criteria should be considered with every new order of clinical systems.

#### Topic 3: Data Provenance—“Trace Your Data, Even Within Large-scale Efforts.”

##### Introduction

Meaningful and standardized metadata facilitate the interpretation of, retrieval of, and access to data [41]. When explainable data are processed with interoperable tools, scientists can create automated and reusable workflows and provide access to reproducible research outcomes and data analysis pipelines [42].

##### Insight

Data provenance describes the history of digital objects, where they came from, how they came to be in their present state, and who or what acted upon them [43]. In health care, provenance maintains the integrity of digital objects (eg, the results of data analyses engender greater trust if their provenance shows how they were obtained). In addition, it can be used to deliver auditability and transparency, specifically, in learning health systems, and it is applicable across a range of applications [44]. Inau et al [45] argued that the lessons learned from “FAIRification” processes in other domains will also support evidence-based clinical practice and research transparency in the era of big medical data and open research. Further work demonstrated that a findable, accessible, interoperable, reusable (FAIR) research data management plan can provide a data infrastructure in the hospital for machine-actionable digital objects [46]. Recently, the openEHR approach was also suggested for creating FAIR-compliant clinical data repositories as an alternative representation [47].

##### Impact

Key data management requirements are defined by the FAIR guiding principles [48]. Since data protection laws led to additional requirements for data privacy and data security, the FAIR-Health principles focused on defining additional requirements for information on the sample material used from biobanks, for provenance information, and incentive schemes [49]. Further work is needed to establish provenance frameworks in health research infrastructures [50].

#### Topic 4: Data Sharing—“If Data Won’t Come to the Model, the Model Must Go to the Data.”

##### Introduction

Cross-sectional medical data-sharing is critical in modern clinical practice and medical research, in which the challenge of privacy-preserving transfer and utility needs to be addressed [51]. In order to facilitate high reuse of the data, a decentralized computational scheme that treats the available data as part of a federated (virtual) database, avoiding centralized data collection, processing, and raw data exchanges, is still needed in many countries to analyze large and widespread clinical data [52].

##### Insight

One possible solution for this federated learning approach is DataSHIELD [53]. In particular, orchestrating privacy-protected analyses of “medical big data'' from different resources is applicable within R and DataSHIELD [54]. Here, the developed computerized models represent mathematical concepts or trained machine learning (ML)–based approaches to solve a specific task. In this sense, the model is applied to distributed data sets of the protected (clinical) server infrastructure, and the user only sees the model results but does not retrieve any medical records. Moreover, implementations in other programming languages (eg, Python, Julia) have been introduced in the genomic domain and beyond [55]. Further concepts, such as Personal Health Train, specifically follow the FAIR principles during distributed analyses [56]. Secure multiparty computation (SMPC) is also a viable technology for solving clinical use cases that require cross-institution data exchange and collaboration [57]. Current limitations are thought to be addressed in a stepwise manner [58] or as blockchain [59].

##### Impact

By using approaches for distributed analyses, researchers are able to train, test, and validate their models on large-scale real-world clinical data. In combination with standardized data formats, these 2 concepts facilitate the use of those models in clinical routine, potentially in the form of a CDSS. This provides a basis for secondary use of observational data in the context of clinical trials, which show particular potential for identifying data characteristics in small cohorts (eg, identification of the individual patient risk for rare diseases or comorbidities).

### Harmonization and Processing

#### Topic 5: Extract, Transform, and Load (ETL)—“ ETL Processes Are Computational Approaches for Data Harmonization and Data Unification.”

##### Introduction

Data handling in medical informatics remains a major challenge. Even though most data in medicine are available electronically, the data often lack interoperability [60]. As a first step to actually use the data, processes to extract, transform, and load (ETL) are needed to obtain harmonized data from different data systems or clinical entities. One important example, among many others, reflects the uniform representation of the date and time in a common format (eg, Year-Month-Date, not Date-Month-Year). The ETL process is therefore a crucial, individual step toward data unification in large clinical systems, which must be secure, safe, and accurate [61].

##### Insight

The design of an ETL process faces several challenges, including the following: (1) The ETL process should be able to process huge amounts of data at once [62]; (2) the ETL process should be repeatable—if the source data change, the ETL process needs to be rerun to process the source data (Observational Health Data Sciences and Informatics [OHDSI]) [63]; (3) expert-level anonymization methodologies might be integrated into ETL workflows whenever possible [61]; and (4) there is a need to check for loss of data and compromised data integrity. The latter was highlighted in a recent study, in which inaccurate cohort identification took place because erroneous vocabulary mappings of a common data model were used (eg, ETL programming bugs and errors not captured during the quality assurance stages) [64]. Common solutions to implement ETL processes are code-based (eg, FHIR-to-OMOP [65]) or via Pentaho Data Integration, which is one of many ETL tools. Further subsequent processing may also include loading data into research data repositories, like OMOP (see Topic 7), tranSMART, and Talend Open Studio, which is a central component of the Integrated Data Repository Toolkit [66].

##### Impact

Since ETL processes are at the core of data handling, all risks associated with the ETL process need to be thoroughly checked, identified, and assessed, and contingency plans to mitigate these risks should be in place [67]. Once the ETL processes are executed, the resulting data will be *trusted* by researchers, who heavily rely on comprehensively checked data integrity to be able to conduct their research on this basis.

#### Topic 6: FHIR—“Set FHIR to Gain a Communication Standard for Real-time Applications at the Device-to-Device Level.”

##### Introduction

Interoperability levels can be divided into technical, syntactic, semantic, and organizational interoperability [2]. Semantic and syntactic interoperability can be ensured by communication exchange standards, such as the FHIR [68] standard of Health Level 7 (HL7) and medical terminologies. A suitable starting point for the basic procedures is offered by FHIR drills [69] or fire.ly [70].

##### Insight

FHIR is one of many communication standards but will likely change the domain of clinical IT significantly [71,72]. As a communication standard, FHIR harmonizes data formats coming from different CIS and enables data exchange between institutions via a RESTful approach [73]. Moreover, FHIR is used to connect devices with each other, which means, in particular, that the Integrating the Healthcare Enterprise (IHE) [74] standard has been revised to support HL7 messaging as well. In turn, IHE has been developing an open-source device tool set for home and hospital use that recently enabled device control capabilities, a capability accelerated during the COVID-19 pandemic to allow nurses and physicians to operate ventilators and infusion devices outside the contaminated patient room [75].

##### Impact

Utilizing FHIR in multiple applications already shows its versatile and flexible use (eg, in mobile health applications [76], electrocardiogram monitoring [77], or wearable devices and precision medicine in digital health [72]). In particular, the SMART-on-FHIR technology enables third-party app development for health care applications [78] and encompasses feasible, secure, and time- and resource-efficient solutions [79,80].

#### Topic 7: OMOP—“Use Common Data Models as Well-defined Representations of Large-scale Research Projects.”

##### Introduction

Data harmonization enables research teams to run real-world observational studies based on heterogeneous data across country borders. Thus, harmonized data embedded in a common data model (CDM), which is an agreement about the utilization of standardized terminologies for data representation, is crucial to exchange data and results on a large scale. To foster reliability and trust in the results of observational research on real-world data, it is essential to utilize CDMs whenever possible to ensure a high degree of data analysis reproducibility.

##### Insight

Several CDMs exist for that purpose; the OMOP CDM from the OHDSI community is one of the most promising and established approaches. In comparison with other CDMs, such as the Sentinel CDM or Informatics for Integrating Biology and the Bedside (i2b2), the OMOP CDM has broader terminology coverage [81]. The importance of the OMOP CDM increased a lot over the last years [82], not least since the European Medicines Agency initiated the Data Analysis and Real World Interrogation Network (DARWIN) [83] project to establish a research network in Europe to gain real-world evidence based on OMOP. Moreover, representations of genomic data [84], oncology [85], and imaging projects [86] are also suitable. In addition, the common representation of the data in OMOP semantic interoperability is ensured by utilizing international terminologies and vocabularies, such as SNOMED-CT, the International Statistical Classification of Diseases and Related Health Problems (ICD), the Logical Observation Identifiers Names and Codes (LOINC), and RxNorm to represent every clinical fact in OMOP. Additionally, the open-source OHDSI software stack provides standardized methodology and libraries for data analyses (Athenahene, Atlas, HADES) and training (EHDEN Academy) [87], as well as a framework to assess and improve data quality to foster reliability and trust in research results [88].

##### Impact

The OMOP CDM is one possibility to represent and analyze clinical data on a research scale. Definition of new cohorts within OMOP enables researchers to quickly investigate questions spanning multiple research entities. Collectively, both FHIR and OMOP can define the structure and relations of the clinical data corpus, and the individual EHRs provide content to these standardized data reservoirs. In comparison, OMOP is commonly used for static large-scale data analysis of research data, and FHIR is more suitable for rapid data integration scenarios (ie, for real-time applications and analysis). In summary, it is important to know and utilize newly established standards to participate in broader clinical networks for research. This way, all information within the EHR is comparable across different clinical sites and research settings.

### Evaluation, Visualization, and Dissemination

#### Topic 8: Data Quality—“Guarantee High Quality and Then Publish the Data.”

##### Introduction

What is meant to be appropriate data quality for health informatics research? In this domain, data quality depends on the quality of single data elements, data completeness, data conformance, and data plausibility aspects that may considerably determine the validity and veracity of analysis results [89,90]. Moreover, data quality across different institutional entities and even health sectors requires additional efforts concerning the different personnel, instruments, and more [91]. High-quality data at hand is one fundamental requirement that is often difficult or impossible to achieve, which is why the generation of synthetic data can be an alternative that satisfies privacy problems as well as research needs when data are expensive, scarce, or unavailable by augmentation [92].

##### Insight

First, a major problem is that clinical data have to be electronically recorded, accessed, and standardized in order to run quality assessment processes [26]. In addition, it would be important to design and use the same data quality tool, standard operating procedures, or ETL mapping rules in all involved institutions. However, in real-life scenarios, there is a lack of both centrally coordinated data quality indicators and formalization of plausibility rules, as well as a repository for automatic querying of the rules, especially in ETL processes [93]. Although numerous data quality evaluation frameworks exist, no clear and widespread approach has been adopted so far [67,94-96]. Even after a well-chosen data quality procedure is properly implemented, clinical data as such cannot be published along with the performed study. As an alternative, synthetic data generation models function in the following 2 different ways: (1) The model is trained, for example, using real-world data and, once trained, will not require any data in the future (model-based approaches), and (2) the model is constantly fed with data to generate synthetic data (data-driven approaches). There are 3 different categories of algorithms used in the generation of synthetic data: probabilistic models, such as Bayesian networks [97] and Copulas [98]; ML, such as Classification and Regression Trees (CART); and deep learning methods, such as a generative adversarial network (GAN) [99-101] and variational autoencoder (VAE) [102].

##### Impact

A combination of appropriate data quality evaluation and synthetic data generation highly facilitates the development of accurate AI models, which are essential in medical studies [103]. Thus, a corpus of high-quality synthetic data with many patients can be reused by other AI experts for model development and benchmarking. Moreover, it is essential to create an infrastructure that is used across a large community of hospitals; maps the entire treatment process electronically; and only generates interoperable, structured data based on FHIR (Topic 6) and OMOP (Topic 7) in accordance with the FAIR principles (Topic 3). Afterward, one can finally run quality assessment processes.

#### Topic 9: Clinical Decision Support Systems—“Bring Insights, Not Additional Work, Back to the Clinics via a CDSS and Other User-Centric Applications.”

##### Introduction

CDSS are computer systems designed to assist the medical staff with decision-making tasks about individual patients and based on clinical data [104]. The decision-making process is still, and will remain, on the shoulders of the physician [105]. The categories of CDSS include knowledge-based systems that make use of clinical rules, nonknowledge-based systems (eg, AI-based systems), and hybrid CDSS that likewise utilize clinical models and knowledge in combination with AI.

##### Insight

The use of a CDSS in a well-implemented clinical workflow has many positive aspects. It may lead to fewer error rates [106], accelerate rare disease diagnosis [107], increase radiologists’ job satisfaction [108], offer personalized cancer treatment [109], or help with real-time cardiovascular risk assessment [110]. Interestingly, computerized alerting systems, which are one of the most disseminated CDSS, can decrease drug-drug interactions significantly [111]. On the other hand, if done improperly, a CDSS can cause alert fatigue by creating too many alerts. If a system is not context-sensitive, alerts can even be inappropriate [112]. According to Olakotan et al [112], influencing factors of a well-designed CDSS need to include aspects about the (1) technology (eg, usability, alert presentation, workload, and data entry), (2) human (eg, training, knowledge, skills, attitude, and behavior), (3) organization (eg, rules and regulations, privacy, and security), and (4) and process (eg, waste, delay, tuning, and optimization). To avoid a lack of transparency and facilitate acceptance by physicians, especially with nonknowledge-based systems, current CDSS seek to use explainable AI approaches; however, the selection of methods used to present explanations in an informative and efficient (*clinically useful*) manner remains challenging [113]. Of note, a CDSS may also have a negative influence on the performance of physicians, especially if inadequate suggestions occur more often, which cannot be compensated with explanations [114]. However, one among many other prominent approaches to obtain such explanations via ML-based feature selection and ranking can be found in the work from Wolfien et al [115]. In terms of an OMOP-based implementation in research, there is patient-level prediction (PLP), which is designed to foster the clinical decision-making process concerning diagnoses or treatment pathways based on the EHR of the patient and the current clinical guideline. It is used to answer questions, such as identifying patients among a larger population at higher risk of a certain outcome (eg, occurrence of cancer, severe side effects, or death) by using data in standardized formats (eg, as previously described via OMOP CDM). Once the model is designed, the covariates will be extracted from the respective CDM of the target person within the cohort, and the respective outcome will be predicted (eg, via PLP [116,117] or other customized prediction algorithms). Importantly, the results from model prediction should first be internally validated with previously unseen data and afterward compared with established scoring systems (eg, Framingham Risk Score [118], SCORE2 [119]) to connect with already known domain-specific contexts and to prove its benefit in clinical practice. An additional validation with external data, as part of a multicenter study, can be seen as highly beneficial, in which the already presented topics of federated learning (Topic 4) and OMOP (Topic 7) could significantly foster such an essential scenario [120].

##### Impact

Collectively, a CDSS increases patient safety, assists in clinical management, and can be cost-effective [104]. In general, findings of even erroneous CDSS can be used to guide the design of new CDSS alerts. However, the existing risks cannot be solved solely on a technical basis and require an interdisciplinary effort. In particular, continuous, clear communication between IT professionals (developers) and health professionals (end users) during the design process is key. Only a profound understanding of the needs and requirements of either of the involved parties can lead to well-designed systems that are actually able to support and relieve physicians in doing their job.

#### Topic 10: Visualizations—“Improved Dissemination of Local and External Data From Computational Models by Well-defined Interactive Visualizations.”

##### Introduction

Large volumes of data collected from patient registries, health centers, genomic databases, and public records can potentially improve the efficiency and quality of health care via enhancing the interoperability of medical systems, assisting in clinical decision-making, and delivering feedback on effective procedures [121]. However, each and every raw data point must go through different analytical processes until they become useful and interpretable at the point of care.

##### Insight

R and Python are 2 versatile open-source programming languages that have gained popularity for different purposes, such as preprocessing (eg, tidyverse), statistical tests (eg, dplyr), ML and deep learning (eg, mlr package, caret), visualization (eg, ggplot), and writing reports directly using knitr and R markdown (RStudio education [122]). Like R, Python offers different libraries for data science tasks (eg, open mined [123]) in addition to a library specifically for health predictive models, namely PyHealth [124]. Another versatile visualization functionality is offered for both languages via R Shiny [125] and Plotly Dash [126]. These 2 platforms enable data scientists to create interactive web applications directly from a script. The applications can be extended using embedded CSS themes, HTML widgets, and Javascript actions. There is already evidence that implementing clinical dashboards or CDSS for immediate access to current patient information can improve processes and patient outcomes [127], especially if the data sets are further evaluated and refined [128]. Similar to FHIR, OHDSI provides tools for analyzing data in the OMOP CDM, which are written in R and use Shiny for the visualization. As a plus, data already stored in the OMOP CDM format can be used in systematic studies, patient-level analysis, and population-based estimations from scratch. The cBioPortal is one prime example of a web resource for exploring, visualizing, and analyzing multidimensional data, which reduces molecular profiling data from cancer tissues and cell lines into readily understandable genetic, epigenetic, gene expression, and proteomic events [129]. It was recently demonstrated how cBioPortal can be extended and integrated with other tools to a comprehensive and easily deployable software solution that supports the work of a molecular tumor board [130] and even deliver meaningful scientific insights [131]. Another translational research platform for the construction and integration of modern clinical research charts is Informatics for i2b2, which is also at the heart of clinical research [132,133].

##### Impact

Computational approaches and data analyses are tightly connected with medical research; the visualization of such complex data for clinicians in a routine setting especially plays a larger role. The current developments of translational research platforms, such as cBioPortal and i2b2, enable swift translation of research results into the clinic, if adequately adopted and enough trained people supervise the process.

## Discussion

### Overview

**Figure 2:**
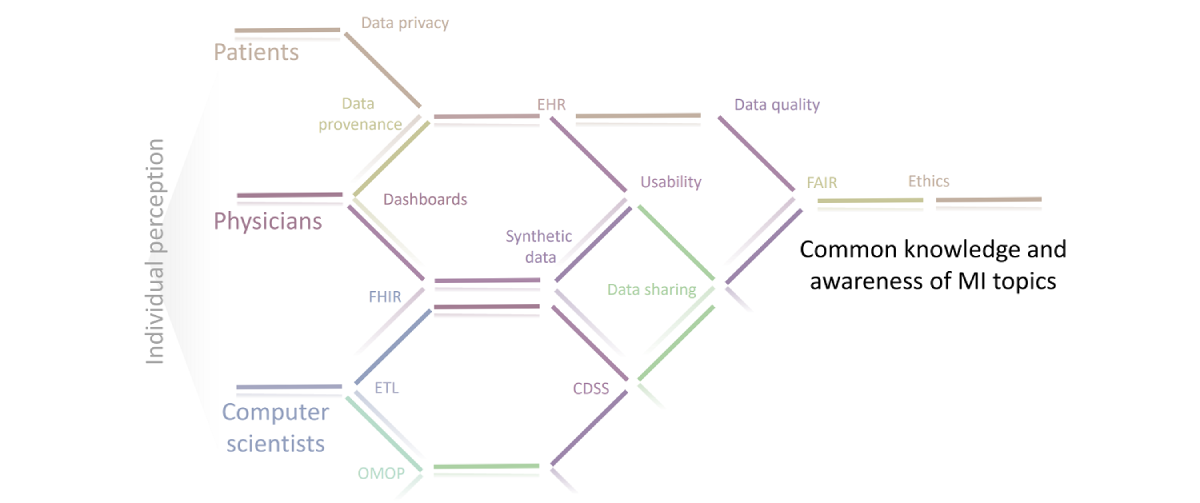
Exemplary outcome visualization of the underlying study, in which the color coding reflects the initial colors of the proposed sections; it starts with an individual perception of the term medical informatics (MI) based on the individual’s background and ends with acquisition of common domain knowledge for current important topics. CDSS: clinical decision support system; EHR: electronic health record; ETL; extract, transform, and load; FAIR; findable, accessible, interoperable, reusable; FHIR; Fast Healthcare Interoperability Resources; OMOP: Observational Medical Outcomes Partnership.

The need for qualified IT specialists in medical informatics has increased continuously in recent years and will continue to grow in the future. On the other hand, medical informatics in Germany faces problems with the ​​promotion of young researchers. These current developments mean that vacancies in IT in hospitals and the health care industry can often not be filled or only after very long vacancies. In addition, these positions often have to be filled with nonspecialist staff due to a lack of applications. To keep track of these recent developments and provide a basis for interdisciplinary communication, we provide our list of 10 topics that could be used by different stakeholders individually (Figure 2). With a particular focus in medicine, improved interdisciplinary communication has already been shown to positively impact patient outcomes and enhance employee engagement [134].

Furthermore, medical informatics has developed rapidly in recent years. This applies, for example, to new methods, techniques, tools, framework conditions, and organizational structures, especially in the field of medical data science. In particular, definitions of standards and a national digitized data corpus, namely the German Core Dataset [135], were agreed upon. The actual assessment and collection of digitized data in local university hospitals are utilized in so-called data integration centers. These interoperable research data infrastructures enable rapid multisite research, for example, with complex COVID-19 research data sets (German Corona Consensus Dataset [GECCO]) [136] including clinical data and data on biosamples from all German university hospitals in pseudonymized form (CODEX) [137,138] or the COVID-19 Data Portal [139]. The subsequent formation of the Network University Medicine (NUM) strengthens the existing interaction between research and patient care, stabilizes existing structures, and creates new structures that ensure more effective feedback and close cooperation between the clinics. The presented examples of NUM and CODEX, among others [140], attempt a central approach to bundle and harmonize necessary resources like broad consent or the elektronische Patientenakte (ePa), which is the implementation of EHR as a national entity to ultimately facilitate an interconnected health care system.

Finally, all those involved in medical informatics are called upon to engage in lifelong learning and continuously acquire further qualifications.

### Exemplary Implementation of the Addressed Topics in the German Medical Informatics in Research and Care in University Medicine Consortium

This article offers newcomers to medical informatics a first introduction and a wealthy overview of current IT-related topics in research and patient care. Nevertheless, there is also a need for further qualification of employees through new, innovative offers for training, further education, and further training. As part of the MII [11], all consortia were asked to develop and set up appropriate offers and formats. The Medical Informatics in Research and Care in University Medicine (MIRACUM) consortium [141] has reacted and set up the part-time training and further education program “Biomedical Informatics and Data Science” [142] and introduced it at the Mannheim University of Applied Sciences in October 2020. The program includes a time-flexible and individually adaptable part-time online master’s course, as well as certificate courses and programs for further scientific education. In addition to the establishment and continuous further development of a cloud-based learning platform, many new digital and target group–oriented learning resources and application-oriented learning environments were developed and introduced for the master's program.

All 10 topics listed in this article are reflected in the curriculum of the master’s degree and have been offered and dealt with in-depth in the individual courses for more than 2 years. The demand for the master’s program and certificate courses is high, and the evaluation has shown that these topic-specific foci correspond to the training and further education needs of the target groups. One particular aspect that was not covered in the final topics refers to the underlying infrastructure needed to provide the data storage and processing backbone. This aspect would have been too technical for a more broadly set, introductory article, such as this article. A starting point for more in-depth information about this aspect can be obtained from further literature [143,144]. However, to offer a practical start to the 10 topics, we provide links to well-known tutorials and hands-on materials (Table 1).

**Table 1:** Summary of tutorials and hands-on material about medical informatics standards and applications.

Topic number	Name	Description	Link
2	SNOMED CT^a^	This 5-step briefing presents a high-level overview of SNOMED CT, how it works, and the benefits of use.	[35]
4	DataSHIELD	This tutorial introduces users to DataSHIELD commands and syntax in R/R Studio.	[145]
5	ETL^b^	This provides introductory material to get from the native/raw data to the OMOP^c^ CDM^d^ one needs to create an ETL process.	[63]
6	FHIR^e^ training	This contains a series of FHIR tutorials for those just beginning to learn the new specification.	[70]
6	SMART App Gallery	The SMART platform is composed of open-standard, open-source tools for developers building apps, and a publicly accessible gallery.	[78]
7	EHDEN Academy	This contains a series of tutorials for OMOP CDM and additional OHDSI^f^ tools (eg, PLP^g^ [117]).	[87]
8	Synthetic data generation	This is a hands-on tutorial from the ODI^h^ [146] showing how to use Python to create synthetic data	[147]
10	R Studio education	This provides an introduction to basic R programming.	[122]
10	Python Dash	This tutorial helps develop data visualization interfaces.	[148]

^a^SNOMED CT: Systematized Nomenclature of Medicine and Clinical Terms.

^b^ETL: extract, transform, and load.

^c^OMOP: Observational Medical Outcomes Partnership.

^d^CDM: common data model.

^e^FHIR: Fast Healthcare Interoperability Resources.

^f^OHDSI: Observational Health Data Sciences and Informatics.

^g^PLP: patient-level prediction.

^h^ODI: Open Data Institute.

### Conclusion

We suggest a set of 10 topics to ease the start for researchers and clinicians to become engaged with basic concepts in health informatics research. We provide current review articles for more in-depth reading about the specific topic and present practical hands-on material. The presented topics likewise serve as a broad overview of the medical informatics research domain but also guide individuals and their specific interests. For example, a computer scientist familiar with CDSS development could more easily connect with important aspects, such as data privacy, FHIR, and specific EHRs that are highly relevant for daily work. In contrast, medical experts can obtain an overview of behind-the-scenes technologies, like ETL processes and underlying data quality approaches that are finally visualized as a summarizing clinical dashboard. For readers, we provided a first step toward an improved understanding of a lively and quickly expanding field, but more novel technologies and practical knowledge are ahead. Suggestions and contributions to improve the current topics can be made at GitHub, which will likewise enable content and readers to stay current [12].

## Abbreviations

AI: artificial intelligence
CART: Classification and Regression Tree
CDM: common data model
CDSS: clinical decision support system
CIS: clinical information system
DARWIN: Data Analysis and Real World Interrogation Network
EHR: electronic health record
ePa: elektronische Patientenakte
ETL: extract, transform, and load
FAIR: findable, accessible, interoperable, reusable
FHIR: Fast Healthcare Interoperability Resources
GAN: generative adversarial network
GDPR: General Data Protection Regulation
GECCO: German Corona Consensus Dataset
HL7: Health Level 7
i2b2: Informatics for Integrating Biology and the Bedside
ICD: International Statistical Classification of Diseases and Related Health Problems
IHE: Integrating the Healthcare Enterprise
LOINC: Logical Observation Identifiers Names and Codes
MII: Medical Informatics Initiative
MIRACUM: Medical Informatics in Research and Care in University Medicine
ML: machine learning
NUM: Network University Medicine
OHDSI: Observational Health Data Sciences and Informatics
OMOP: Observational Medical Outcomes Partnership
PLP: patient-level prediction
SMPC: secure multiparty computation
SNOMED CT: Systematized Nomenclature of Medicine and Clinical Terms
VAE: variational autoencoder
